# Shikonin Induces Apoptosis, Necrosis, and Premature Senescence of Human A549 Lung Cancer Cells through Upregulation of p53 Expression

**DOI:** 10.1155/2015/620383

**Published:** 2015-02-09

**Authors:** Yueh-Chiao Yeh, Tsun-Jui Liu, Hui-Chin Lai

**Affiliations:** ^1^Department of Natural Biotechnology, Nanhua University, Sec. 1, No. 55, Nanhua Road, Dalin, Chiayi 62249, Taiwan; ^2^Cardiovascular Center and Department of Anesthesiology, Taichung Veterans General Hospital, Sec. 4, No. 1650 Taiwan Boulevard, Taichung 40705, Taiwan; ^3^Department of Medicine and Cardiovascular Research Center, National Yang-Ming University School of Medicine, Sec. 2, No. 155, Linong Street, Taipei 11221, Taiwan

## Abstract

Shikonin, a natural naphthoquinone pigment isolated from *Lithospermum erythrorhizon*, has been reported to suppress growth of various cancer cells. This study was aimed to investigate whether this chemical could also inhibit cell growth of lung cancer cells and, if so, works via what molecular mechanism. To fulfill this, A549 lung cancer cells were treated with shikonin and then subjected to microscopic, biochemical, flow cytometric, and molecular analyses. Compared with the controls, shikonin significantly induced cell apoptosis and reduced proliferation in a dose-dependent manner. Specially, lower concentrations of shikonin (1–2.5 *μ*g/mL) cause viability reduction; apoptosis and cellular senescence induction is associated with upregulated expressions of cell cycle- and apoptotic signaling-regulatory proteins, while higher concentrations (5–10 *μ*g/mL) precipitate both apoptosis and necrosis. Treatment of cells with pifithrin-*α*, a specific inhibitor of p53, suppressed shikonin-induced apoptosis and premature senescence, suggesting the role of p53 in mediating the actions of shikonin on regulation of lung cancer cell proliferation. These results indicate the potential and dose-related cytotoxic actions of shikonin on A549 lung cancer cells via p53-mediated cell fate pathways and raise shikonin a promising adjuvant chemotherapeutic agent for treatment of lung cancer in clinical practice.

## 1. Introduction

Lung cancer remains a major challenge to global public health for the high epidemiologic incidence and the often-catastrophic outcome at the time of diagnosis [1, 2]. Non-small-cell lung cancer (NSCLC), including adenocarcinoma, squamous cell carcinoma, and large-cell carcinoma, accounts for ~80% of all lung cancers [3] and requires surgical removal as the sole curative therapy which yet could be considered only in a limited proportion of patients at still early disease stages [2]. In most other circumstances the tumor is already advanced and inoperable, and adjuvant pharmacological therapy with or without surgery plays the exclusive role for eradication of malignant cells to achieve complete remission regardless of the underlying pathological types [3]. Nonetheless, contemporary chemotherapy employing various antineoplastic agents either kills malignant cells incompletely or exerts serious systemic adverse toxicity and fatal organ dysfunction. Up to date there is still a lack in a chemotherapeutic agent that could perfectly get rid of all cancer cells without damaging nonmalignant tissues.

Studies have shown that a majority of current chemopreventive and therapeutic approaches for lung cancer could potentially prevent or inhibit its development, proliferation, and invasion [3, 4]. Novel antineoplastic agents intervening in mediators regulating expression of genes involved in tumorigenesis, tumor suppression, and DNA repair are also under rigorous development [5]. Recently, chemicals that potently inhibit cancer growth through various mechanisms, including apoptosis, necrosis, and premature senescence of tumor cells, are under similarly wide survey [6, 7]. Of interest, specified natural herbal medicines proposed promising options for the development of efficient adjuvant chemotherapy for cancer patients [8].

Complementary and alternative medicine, for example, traditional Chinese medicine (TCM), is now widely recognized worldwide [9]. Recent researches have revealed its therapeutic effects in treating various diseases, including diseases [10] and cancer [11]. Shikonin (SHK) is a chief ingredient of the traditional Chinese medicine* Lithospermum erythrorhizon* (purple gromwell) [12] that has been well-known for its multiple biological and pharmacological actions encompassing anti-inflammatory [13, 14], antimicrobial [15], antiadenoviral [16], antiangiogenic [17], antiplatelet [18], wound-healing provocative [19], and proteasome inhibitor [20] activities. It does no harm to normal cells [21] but exerts toxic effects on various neoplastic cells and has not been linked to resistance against anticancer drugs [22]. Its cytotoxic actions are largely through enhancing reactive oxygen species (ROS) generation to trigger caspase-dependent apoptosis and to downregulate nuclear factor-kappa B- (NF-*k*B-) mediated matrix metalloproteinase (MMP) expressions to reduce tumor survival and invasion [23–27]. Specifically, SHK has been demonstrated by* in vitro* and* in vivo* as well as clinical studies to potently trigger apoptosis of lung tumor cells [28–31]. However, no studies have ever specifically evaluated whether p53, a transcriptional factor mediating cellular apoptosis [32], plays a role in cellular senescence contributing to SHK-induced toxicity in lung tumor cells. The present study was therefore designed to investigate whether cytotoxic mechanisms activated by SHK induced p53-mediated apoptosis, necrosis, and cellular senescence in human A549 lung cancer cells. These results of this study could help understand the tumor-suppressive effects of SHK on malignant cells and may hopefully raise this Chinese herbal medicine a potential antineoplastic agent in treatment of the otherwise drug-refractory cancer disease.

## 2. Materials and Methods

### 2.1. Reagents

SHK, DOX, and BLM were obtained from Calbiochem Co. (San Diego, CA, USA), Pfizer Italia S.R.L. (Milano, Italy), and Nippon Kayaku Co. (Tokyo, Japan), respectively. DMEM and FBS were purchased from GIBCO (New York, NY, USA). Propidium iodide (PI) was from Molecular Probe (Eugene, OR, USA). Anti-p53 and cytochrome *c* were purchased from Lab Vision Corp. (Fremont, CA, USA) and anti-*α* tubulin antibodies from Millipore Corporation (Billerica, MA, USA). Horseradish peroxide- (HRP-) conjugated secondary antibodies to mouse, rabbit, and goat immunoglobulins were purchased from Invitrogen (Carlsbad, CA, USA). All other antibodies were from Santa Cruz Biotechnology (Santa Cruz, CA, USA) and chemicals from Sigma (St. Louis, MO, USA).

### 2.2. Cell Culture and Shikonin Treatment

Human non-small-cell lung cancer (NSCLC) cell line A549 cells were obtained from ATCC (#CCL-185) and were cultured in DMEM medium containing 10% heat-inactivated FBS, 2 mM L-glutamine, and 100 *μ*g/mL of penicillin-streptomycin. The cells were maintained in an incubator with a humidified atmosphere containing 95% air and 5% CO_2_ at 37°C and cultured as monolayers and seeded on 10 cm cell culture dishes at a density of 2 × 10^4^ cells/dish. Once reaching 80% confluence, the cells were treated with vehicle (DMSO, 0.1% in media) or SHK (0.5, 1.0, 2.5, 5.0, or 10.0 *μ*g/mL) for the indicated periods to define the anticancer effect of SHK. To investigate the role of p53 in the cytotoxic action of SHK, cells were treated with pifithrin-*α* (45 or 60 *μ*M) 2 hours before the treatment of SHK. The anticancer effect of SHK was then compared to that of two other effective antineoplastic agents, DOX and BLM, at equivalent therapeutic concentrations and duration (24 hours). Changes of cell morphology were observed under the CKX41 inverted phase-contrast light microscope (Olympus, Japan), with the images captured by Spot CCD Camera driven by Advanced Spot RT Software version 3.3 (Diagnostic Instruments Inc., MI, USA). All cells were then scraped down and collected for further molecular and biochemical analyses.

### 2.3. Cell Viability with MTT Assay

Viability of cells in response to SHK treatment was assessed by MTT assay as described previously [33]. Briefly, cells were grown on 24-well culture plates for 4 days prior to treatment. Afterwards, 1 mL of MTT solution (300 *μ*g/mL in 2% FBS/DMEM medium) was added to each well to react at 37°C for 1 hour. The medium was then removed and 300 *μ*L of DMSO was added to each well, pipetted up and down repeatedly to dissolve crystals. Five minutes later, 100 *μ*L of supernatant from each sample was transferred to an ELISA plate for measurement of the absorbance of the converted dye at 570 nm with a reference wavelength of 650 nm. The optical density (O.D.) values reflected the MTT reductase activities and hence the amounts of viable cells.

### 2.4. FACS Analysis for Differentiation of Apoptosis from Necrosis

To analyze the extent of apoptosis and necrosis in response to SHK exposure, cells were trypsinized, pelleted, washed in ice-cold PBS, and resuspended in 1x binding buffer containing Annexin-V-FITC antibody (Invitrogen, Carlsbad, CA) and PI according to the previous study [34] and manufacturer's indicated protocol. The samples were then analyzed by flow cytometry and the emitted fluorescence of the FITC-stained cells excited by a 490 nm argon ion laser was measured at a wavelength of 525 nm in a FACSVantage flow cytometer. Cells were then gated by forward and side scattering. At least 10,000 events were used in calculations for each sample and the proportions of viable, early apoptotic, late apoptotic, and necrotic cells in each sample were estimated from those with low Annexin-low PI, high Annexin-low PI, high Annexin-high PI, and low Annexin-high PI staining, respectively [35].

### 2.5. Caspase-3 Activity Assay

The activity of caspase-3 in A549 cells treated with shikonin was estimated by the capability of this enzyme to cleave the colorimetric substrate (DEVD-pNA for caspase-3) provided in the Caspase-3/CPP32 Colorimetric Assay Kit (BioVision, Mountain View, CA) according to the previous study [5] and following the manufacturer's protocol. Briefly, pelleted cells were harvested using short trypsinization and cell lysates were prepared as described elsewhere [36]. Samples of the cell lysates (50 *μ*g per sample) in 50 *μ*L of chilled reaction buffer were mixed with 50 *μ*L of the 2X substrate working solution and 50 *μ*L of substrate (200 *μ*M final conc.) and incubated for 2 hours at 37°C in the dark. Buffer served as a no-enzyme control to determine the background fluorescence of the substrate. Then, the absorbance was then measured at 405 nm and the sample reading by subtracting the absorbance of blank samples.

Caspase-3 activity was additionally evaluated by flow cytometry using a Casp-GLOW Red-Active Caspase-3 Staining Kit (BioVision, Mountain View, CA, USA), which employs a specific caspase-3 inhibitor DEVD-FMK conjugated with sulforhodamine as the fluorescent marker to allow sensitive detection of activated caspase-3 by flow cytometry. For this method, an aliquot of 300 *μ*L cells was transferred into a microcentrifuge tube and 1 *μ*L of Red-DEVD-FMK was added. Cells were then incubated for 30 min at 37°C with 5% CO_2_, pelleted at 500 g for 6 min, resuspended with 0.5 mL of wash buffer, and centrifuged again. Cells were then resuspended in 300 *μ*L of wash buffer and analyzed using the FL-2 channel of the flow cytometer.

### 2.6. Cellular Senescence Assay

To detect this phenomenon, SA-*β*-Gal was stained using a commercial staining kit with X-gal as the substrate (BioVision, Mountain View, CA) according to the previous studies [6, 37] and following the manufacturer's protocol. Briefly, cells were washed in PBS, fixed in fixative solution for 3–5 min, washed, and incubated at 37°C (without CO_2_) with fresh SA-*β*-Gal stain solution containing 1 mg of 5-bromo-4-chloro-3-indolyl *β*-D-galactoside (X-gal) per mL (stock = 20 mg of dimethyl formamide per mL), 40 mM citric acid/sodium phosphate, pH 6.0, 5 mM potassium ferrocyanide, 5 mM potassium ferricyanide, and 150 mM NaCl/2 mM MgCl_2_. After a 16-hour incubation period, cells were washed with PBS twice and were observed under light microscope. The corresponding digital images were captured for later analysis by a Spot CCD Camera driven by Advanced Spot RT Software version 3.3 (Diagnostic Instruments Inc., MI, USA). The total numbers and the positively stained cells were assessed under microscope at 200x magnification. The senescence index was calculated as the number of positively stained cells/total number of cells × 100%. The average of 5 random observations was taken for each slice.

### 2.7. Immunoblotting Analysis

Cell lysates were prepared following treatment of A549 cells with SHK as described previously [36, 38] and used for immunoblotting analysis. An equal amount of cytosolic protein (20 *μ*g) from each sample was then electrophoretically separated by 12% sodium docecylsulfate-polyacrylamide gel electrophoresis (SDS-PAGE). For the analysis of cytochrome *c*, mitochondrial and cytosolic fractions were prepared from the cells and the cells and the proteins (50 *μ*g) were separated from 15% SDS-PAGE. After being transferred to polyvinylidene difluoride (PVDF) membranes and blocking the nonspecific binding sites, the membranes were incubated with the primary antibodies (1 : 1000 dilutions) at 4°C overnight. After being washed three times with PBS buffer containing 0.1% Tween 20, the membranes were incubated with appropriate HRP-conjugated secondary antibodies (1 : 5000 dilutions) and visualized by enhanced chemiluminescence (ECL) Plus (Pierce, Rockford, IL, USA). Each membrane was stripped and reprobed with anti-*α*-tubulin antibody to ensure equal protein loading. The density of each protein band was scanned using Science Lab 2001 Image Gauge 4.0 Software (Fujifilm, Tokyo, Japan) and compared between groups by densitometry (*α*-tubulin as a reference).

### 2.8. Immunofluorescent Microscopy Analysis

To analyze whether p53 is correlated with release of cytochrome *c* into cytosol from mitochondria, subcellular localizations of these two factors in A549 cells were examined by immunofluorescent microscopy. For this study, cells grown on glass cover slides were fixed with 4% paraformaldehyde in phosphate buffer saline (PBS) for 30 min, washed three times in PBS, and immersed in PBS containing 3% BSA for 1 h to block nonspecific binding. Cells were then incubated with primary antibody at dilutions of 1 : 100 for 18 h at 4°C, washed twice in PBS/0.05% Triton X-100 solution, and reacted with a fluorescein- or rhodamine-conjugated secondary antibody (Invitrogen, Carlsbad, CA) for 1 h at room temperature. Nuclei were stained with DAPI containing mounting medium, and cells were then examined with a fluorescence microscope (Leica DMR, Bensheim, Germany) at a magnification of 400x to determine contents. The corresponding digital images were captured for later analysis by a Spot CCD Camera driven by Advanced Spot RT Software version 3.3 (Diagnostic Instruments Inc., MI, USA).

### 2.9. Statistical Analysis

All data are expressed as the mean ± standard error of the mean (SEM). All experiments were repeated at least 3 times (≥3 replicates) on each specimen and there were 3 specimens from each group. The drug median inhibitory concentration (IC_50_) was calculated by linear-regression models. The results of all replicates from each specimen were averaged, and the mean of averaged values from all specimens of a single group was regarded as the corresponding value of the whole group. Statistical analyses were performed using one-way analysis of variance (ANOVA). Differences between the means of each group in each assay were tested using Dunnet's test. If the mean values of at least one group differed from others with *P* < 0.05, they were considered statistically significant.

## 3. Results

### 3.1. SHK Inhibits Growth of A549 Lung Cancer Cells in a Time- and Dose-Dependent Manner

**Figure 1:**
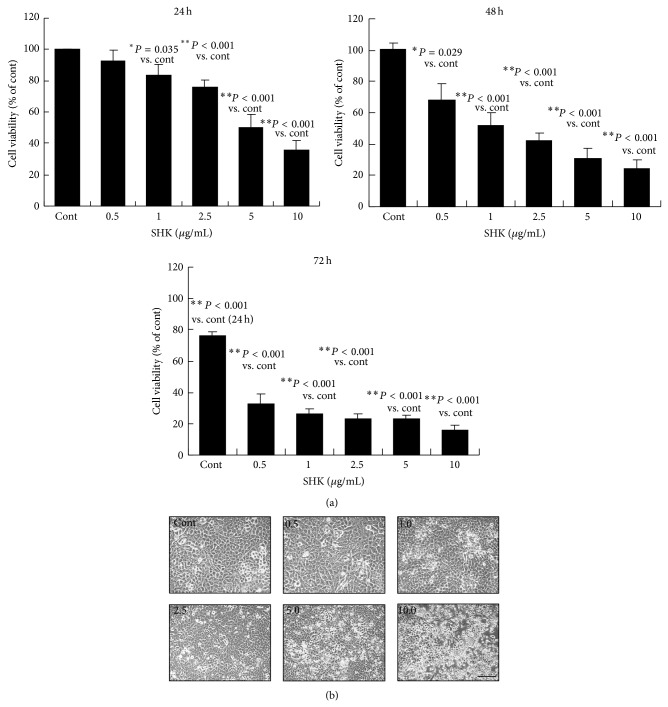
Effects of shikonin on cell proliferation and morphological changes in human A549 lung cancer cells. (a) The cells were treated with vehicle (Cont) or shikonin (SHK) (0.5, 1.0, 2.5, 5.0, or 10.0 *μ*g/mL) for 24, 48, or 72 hours. After indicated periods of treatments, the cell viability was determined by MTT assay. Results were obtained from 3 independent experiments, and the bars represent ± S.E.M. ^*^ *P* < 0.05 and ^**^ *P* < 0.01 compared with differences between the different doses of shikonin at indicated time points, respectively. (b) Representative photomicrographs of A549 cells treated without or with shikonin for 24 hours observed with phase-contrast light microscope. Scale bar indicates 80 *μ*m.

To evaluate the cytotoxic effects of SHK on human lung cancer cells, A549 cells were treated with vehicle or 0.5 to 10.0 *μ*g/mL of SHK for 24, 48, and 72 hours. Cell viability determined by MTT assay (Figure 1(a)) demonstrating SHK inhibited the growth of A549 cells in a time- and dose-dependent manner, and at 72 h of SHK treatment all cell viability decreased to below 30% of the control values. To compare the cytotoxicity of SHK with that of two other conventional chemotherapeutic drugs [39], cells were treated with equivalent therapeutic concentrations of DOX and BLM (0, 0.5, 1.0, 2.5, 5.0, or 10.0 *μ*g/mL) for 24 hours. The 50% inhibitory concentration (IC_50_) of SHK on A549 cells determined by an MTT assay showed that IC_50_ value of SHK (3.52 ± 0.17 *μ*g/mL) was lower than BLM (6.19 ± 0.31 *μ*g/mL) and higher than DOX (0.47 ± 0.02 *μ*g/mL) (Table 1).

Morphology of cells alters in response to external stress as an intrinsic protective mechanism for prevention from death [40]. Phase contrast microscopic study demonstrated that lower-doses of SHK (0.5–2.5 *μ*g/mL) treatment for 24 h reshaped a large number of A549 cells to be distinctly flat and enlarged in size, whereas higher-doses of SHK (5.0 and 10.0 *μ*g/mL) caused reduction of cell numbers, rounding up of cell contours, and shrinkage of cell size with condensed and vacuolated nuclei (Figure 1(b)), indicating the multiplicity of the insulting actions of SHK on A549 cells. These observations showed that SHK has dose- and time-dependent inhibitory effects on the viability of A549 cells.

### 3.2. Effects of SHK on Cell Cycle, Apoptosis, Necrosis, and Cellular Senescence

**Figure 2:**
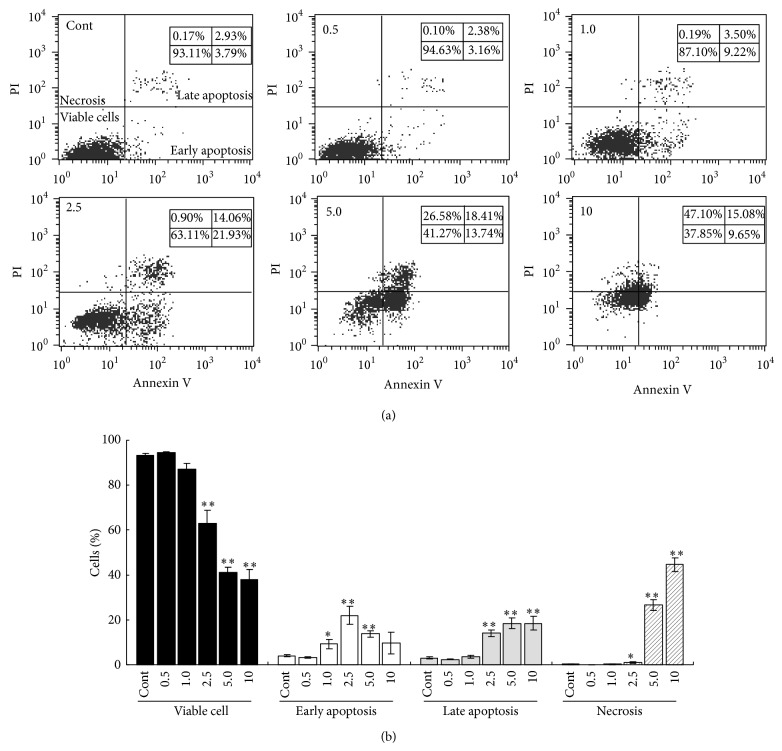
Effects of shikonin on apoptosis and necrosis of A549 cells. (a) Representative flow cytometry results. A549 cells were treated with shikonin (SHK) at different concentrations, stained with Annexin V-FITC/PI and then subjected to flow cytometry. Viable cells (scattered in left lower quadrant of the graphs) decreased whereas early-apoptotic (right lower quadrant), late-apoptotic (right upper quadrant), and necrotic (left upper quadrant) cells increased to various extents in response to SHK treatment. (b) Quantitative results from flow cytometry. Data were represented as mean ± SEM from at least three independent experiments. ^*^ *P* < 0.05, and ^**^ *P* < 0.01 v.s. Cont.

To further investigate whether shikonin cytotoxicity involves apoptosis and necrosis, SHK-treated A549 cells were analyzed by Annexin-V/PI-stained flow cytometry. Overall, proportion of viable cells (low PI, low Annexin V staining) decreased significantly in those treated with SHK at the doses higher than 2.5 *μ*g/mL, as compared to untreated controls (2.5 *μ*g/mL, *P* = 0.002; *P* < 0.001; and *P* < 0.001 versus Cont, resp.) (Figures 2(a) and 2(b)). Specifically, cells started to succumb to early apoptosis (low PI, high Annexin-V staining) when exposed to SHK at doses of 1.0, 2.5, and 5.0 *μ*g/mL, advanced apoptosis (high PI, high Annexin-V staining) at doses of 2.5, 5.0, and 10.0 *μ*g/mL, and necrosis (high-PI/low-Annexin-V staining) at doses of 5 to 10 *μ*g/mL, suggesting that lower doses (1.0 and 2.5 *μ*g/mL) of SHK induce early apoptosis, while higher doses (5.0 and 10.0 *μ*g/mL) of SHK trigger late apoptosis and necrosis of lung cancer cells.

**Figure 3:**
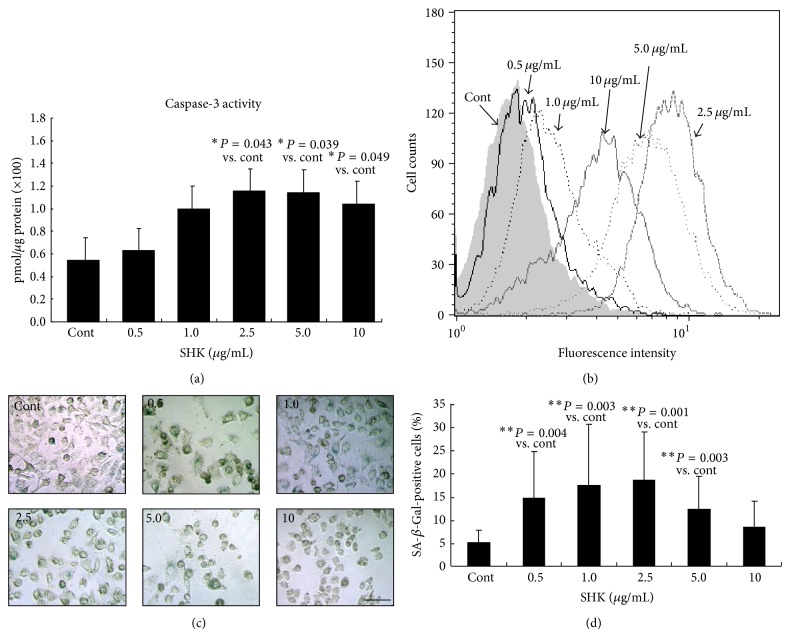
Effects of shikonin on activating caspase-3 cleavage and cellular senescence in A549 cells. (a) Colorimetric analysis. Shikonin (SHK) (2.5 *μ*g/mL) soundly elevated caspase-3 activity by 2.12-fold compared with the Cont. (b) Flow cytometric study. Cells treated with SHK generally expressed higher capsase-3 activity by (peak of capsase-3 activity deviating to the right). (c) Senescence-associated *β*-galactosidase (SA-*β*-Gal) activity. SHK-treated cultured cells contained more positive cells (green color) compared with the Cont by SA-*β*-Gal staining. Scale bar indicated as 20 *μ*m. (d) Quantitative analysis of cellular senescence from SA-*β*-Gal staining. The ratio of positive labeling cells was significantly higher in shikonin-treated cells. Values were obtained from three independent experiments and represented as mean ± S.E.M. Significant difference is indicated by ^*^ *P* < 0.05 and ^**^ *P* < 0.01 versus Cont.

To determine which apoptotic pathway SHK operated to trigger cellular apoptosis, caspase-3 activity was examined and found markedly upregulated by colorimetric (Figure 3(a)) and flow cytometric (Figure 3(b)) analyses in cells treated with SHK at the dose higher than that of 2.5 *μ*g/mL (2.5 *μ*g/mL, *P* = 0.043; 5.0 *μ*g/mL, *P* = 0.039; and 10.0 *μ*g/mL, *P* = 0.049 versus Cont, resp.), indicating the role of caspase-3 in the apoptotic actions of SHK on A549 cells.

Premature senescence represents an indicator of cell dysfunction. As shown in Figure 3(c), when A549 cells were treated with SHK at a concentration of as low as 0.5 *μ*g/mL, typical features of cellular senescence emerged, including flattened and enlarged morphology along with molecular markers of senescence-associated heterochromatin foci and lipofusion granules [37, 41]. Quantitative analyses showed that proportion of cells positive for SA-*β*-Gal activity, another molecular marker of cellular senescence [32, 35], peaked at 0.5 *μ*g/mL of SHK but declined as the doses were increased (14.7%, 17.5%, 18.7%, 12.4%, and 8.6% at doses of 0.5, 1.0, 2.5, 5.0 and 10.0 *μ*g/mL) (Figure 3(d)), depicting the converse relationship between cellular senescence and SHK concentrations above 0.5 *μ*g/mL.

### 3.3. SHK Regulates Expressions of Cell Cycle and Apoptosis-Regulating Proteins

**Figure 4:**
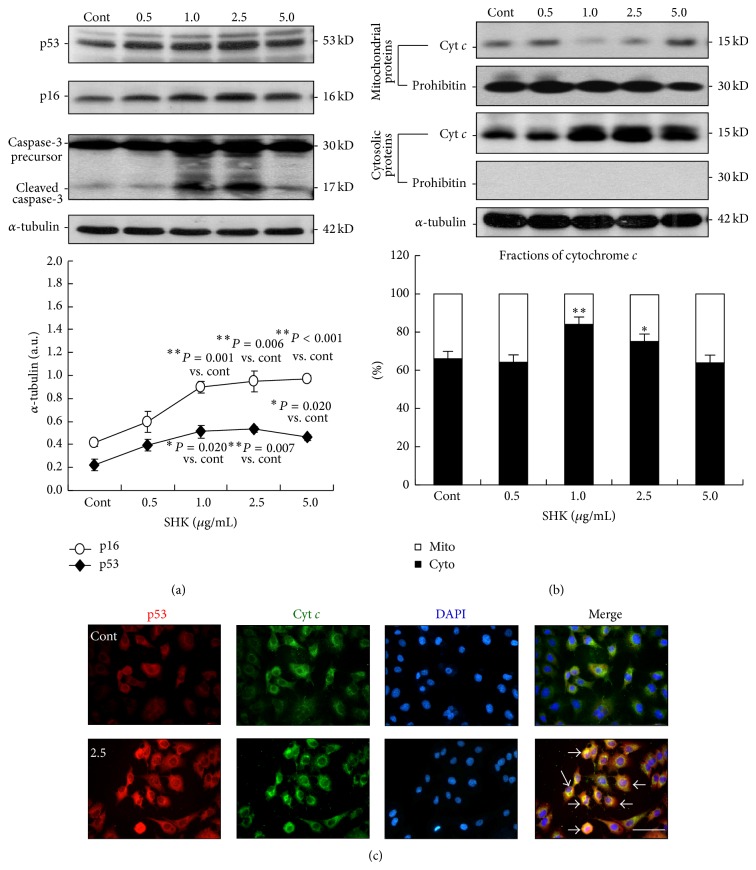
Effects of shikonin on regulation of cell cycling and apoptotic signaling. (a) Immunoblotting. Upper panel, representative immunoblots of p53, p16, and caspase-3 expressions in A549 cells in response to SHK treatment. The level of cleaved caspase-3, a product and marker of caspase-3 activation, was markedly higher in SHK-treated cells (1.0 and 2.5 *μ*g/mL of shikonin). Lower panel, quantitative results from densitometry. Data are from three independent studies. p53 and p16 in cellular extracts of shikonin (SHK) were markedly increased by 549 cells in a dose-dependent manner. Data are also shown as mean ± S.E.M. (*n* = 3 in each group). (b) Upper panel, immunoblotting study of mitochondrial and cytosolic cytochrome *c*. The ample presence of a specific mitochondrial marker, prohibitin, in the mitochondrial extracts (lane 2) and absence of this marker in the cytosolic fractions (lane 4) demonstrates the relative purity of both fractions. The abundance of cytochrome *c* was strikingly decreased in the mitochondria (lane 1) and increased in the cytosol (lane 3) in response to SHK treatment, indicating the triggering effect of SHK on cytochrome *c* release from mitochondria to cytosol. Lower panel, summary data of the cellular distribution of cytochrome *c* in SHK-treated A549 lung cancer cells (1.0 *μ*g/mL versus Cont, *P* = 0.0005; 2.5 *μ*g/mL versus Cont, *P* = 0.018; and mean ± S.E.M., *n* = 3). Cyto, cytosolic fraction of cytochrome *c*; mito, mitochondrial fraction of cytochrome *c*. (c) Immunofluorescent microscopy. Cellular distribution of p53 and cytochrome *c* proteins in A549 lung cancer cell treated with shikonin at 2.5 *μ*g/mL. Red signal and green fluorescence indicate the locations of p53 and cytochrome *c*, and blue color represents the cells counterstained with DAPI. Scale bar indicated as 20 *μ*m.

To determine the mechanism through which SHK triggered apoptosis and cellular senescence, expressions and immunolocalization of cell cycle-related and apoptosis-mediating proteins were investigated. Abundance of p53 assessed by immunoblotting study was obviously increased in cells treated with SHK in a dose-related manner (1.7-, 2.2-, 2.3-, and 2.0-fold of Control at doses of 0.5, 1.0, 2.5, and 5.0 *μ*g/mL), while p16 expression increased in response to 0.5 to 1.0 *μ*g/mL of SHK but significantly decreased at higher doses (1.4-, 2.1-, 2.3-, and 2.3-fold of Control at doses of 0.5, 1.0, 2.5, and 5.0 *μ*g/mL) (Figure 4(a)). Besides, extent of capase-3 cleavage increased significantly in cells treated with SHK at the doses of 1.0 and 2.5 *μ*g/mL (Figure 3(c)) but not at higher doses, suggesting that SHK activated caspase 3-mediated apoptotic signaling in a dose-dependent manner in A549 lung cancer cells.

Immunoblotting analysis of protein fractions from mitochondria and cytosols further demonstrated that the cytosolic fraction of cytochrome *c* (cyt *c*) increased from 66.1% of the total contents (cytosolic plus mitochondrial) in the controls to 84.0% and 75.1% in cells treated with SHK at the doses of 1.0 and 2.5 *μ*g/mL, depicting that SHK induced redistribution of cyt *c* from mitochondria to cytosols (Figure 4(b)). Besides, both p53 and cyt *c* were markedly upregulated and colocalized in SHK-treated cells (at the dose of 2.5 *μ*g/mL) as examined by immunofluorescence analyses (Figure 4(c)), illustrating the apoptotic pathways in A549 lung cancer cells. These results suggest that mitochondrion-dependent, caspase 3-mediated cellular apoptosis, was activated by SHK at the doses of 1.0 to 2.5 *μ*g/mL.

**Figure 5:**
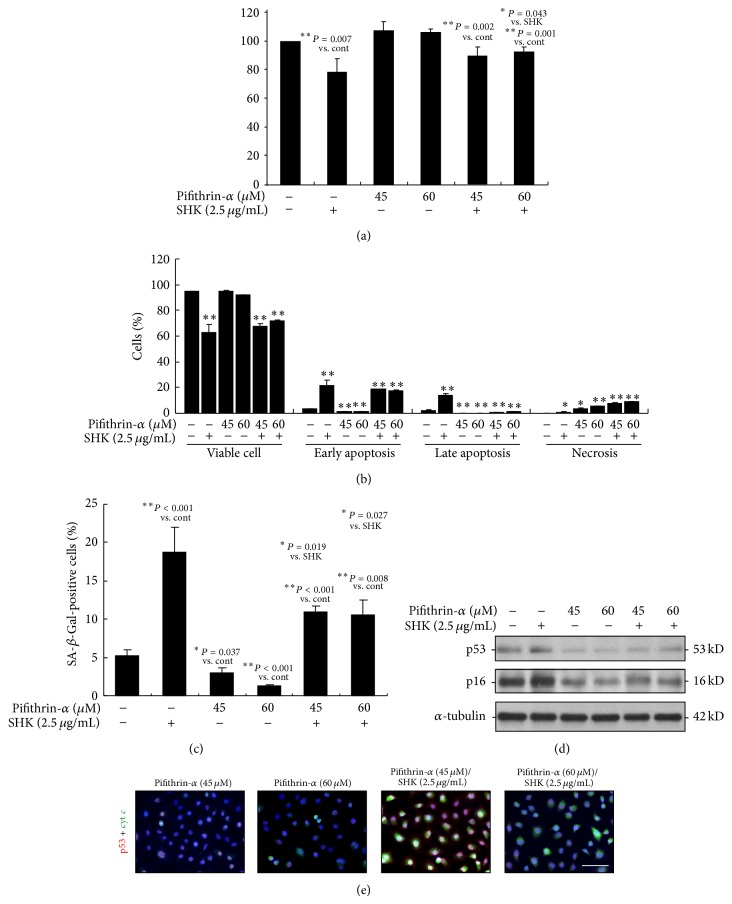
Mediating effect of p53 in shikonin-induced cell growth inhibition, apoptosis, necrosis, senescence, and apoptotic-regulatory protein expressions. (a) MTT study. Pretreatment of A549 cells with pifithrin-*α* (45 and 60 *μ*M) decreases shikonin (SHK) (2.5 *μ*g/mL-) induced inhibition of cell proliferation. (b) Flowcytometric study. Pretreatment of A549 cells with pifithrin-*α* (45 and 60 *μ*M) blocks SHK-induced apoptosis and provokes necrosis after 24 hours of treatment. (c) Quantitative analysis of cellular senescence from SA-*β*-Gal staining. The ratio of positive labeling cells was significantly inhibited by pifithrin in shikonin-treated cells by quantitative analysis of cellular senescence in cells. Values were obtained from three independent experiments and represented as mean ± S.E.M. Significant difference is indicated by ^*^ *P* < 0.05 and ^**^ *P* < 0.01 versus Cont. (d) Immunoblotting analysis. Expression of p53 and p16 significantly increased by shikonin was ameliorated by the p53 inhibitor pifithrin-*α*. Representative sets of data are shown from three independent immunoblotting analyses. (e) Immunofluorescent microscopy. Cellular distribution of p53 and cytochrome *c* proteins in A549 lung cancer cell pretreated with pifithrin prior to administration of shikonin at 2.5 *μ*g/mL. Red signal and green fluorescence indicate the locations of p53 and cytochrome *c*, and blue color represents the cells counterstained with DAPI. Scale bar indicated as 20 *μ*m. These results indicated that the effect of SHK on A549 cells was mediated at least partially by cellular p53.

To determine whether p53 is involved in SHK-induced inhibition of proliferation and induction of apoptosis, necrosis, and senescence on A549 cells, we pretreated the A549 cells with pifithrin-*α* (45 and 60 *μ*M), a specific inhibitor of p53, prior to the treatment with SHK. As shown in Figure 5(a), when treated with SHK (2.5 *μ*g/mL), viability of cells was higher in those pretreated with pifithrin-*α* (94.9% at 45 *μ*M and 91.5% at 60 *μ*M of pifithrin-*α*) compared to those unpretreated (80.1%) indicating the mediating role of p53 in the inhibitory effect of SHK. Pretreatment of pifithrin-*α* also significantly ameliorated SHK-induced apoptosis of the A549 cells (19.4% at 45 *μ*M and 19.0% at 60 *μ*M of pifithrin-*α* versus 36.0% without the treatment of pifithrin-*α*), whereas inhibition of p53 by pretreatment of pifithrin-*α* augmented SHK-induced necrosis (Figure 5(b)). The proportion of positive SA-*β*-Gal stained cells induced by SHK was significantly limited by pretreatment of pifithrin-*α* (Figure 5(c)), hinting the role of p53 in SHK induced cellular senescence. Further, we checked the efficiency of pifithrin-*α* on the status of p53 and p16 in these cells (Figure 5(d)). Immunoblotting analysis revealed that the levels of both p53 and p16 were decreased after the treatment of A549 cells for 24 hours with pifithrin-*α*, and the increased expressions of p53 and p16 induced by SHK were also suppressed by the pretreatment of pifithrin-*α*. Moreover, both SHK-induced upregulations of p53 and cyt *c* were markedly inhibited in pifithrin-*α*-treated cells (Figure 5(e)).

## 4. Discussion

In this study, we have for the first time demonstrated that SHK impedes survival and induces apoptosis, necrosis, and senescence of A549 cells in a dose-dependent manner mediated by cytosolic p53. These findings concur with the results of previous studies demonstrating that SHK can inhibit proliferation of two cancer cell lines [42, 43] and activate p53 activity in various cancer cells [32] and may shed light on employment of this chemical to clinical therapy for such malignant diseases in the future.

### 4.1. Growth-Inhibitory Actions of SHK on Lung Cancer Cells

Lung cancer is among one of malignant diseases that carry the most ominous prognosis if at advanced stages. Though surgical treatment has undergone fundamental changes in the past two decades [4], chemotherapeutic drugs represent the only hope to eradicate residual/metastatic cancer cells in most situations, yet remaining far below satisfaction for the limited overall effectiveness and the potential toxic side effects [39]. Our study exploring the antineoplastic effect of a herb-derived chemical, SHK, against lung cancer cells demonstrated that this novel chemical could exert potent cytotoxic actions on A549 lung cancer cells (IC_50_ value was 3.52 *μ*g/mL for 24-hour incubation) via induction of senescence and apoptosis at relatively lower doses (0.5 to 2.5 *μ*g/mL) and initiation of necrosis at higher doses (5 to 10 *μ*g/mL). These results indicated the potential and dose-related cytotoxicity of SHK against lung cancer cells and implicated SHK a possible adjuvant chemotherapeutic agent for treatment of lung cancer in clinical practice.

SHK has been proven to possess multiple biological activities [10–15], and its cytotoxic activities on various tumors have also been widely reported [23, 24, 28, 30, 44]. One study indicated that low dose of SHK (4 *μ*M) (around 1.33 *μ*g/mL) did not influence the survival of hepatocellular carcinoma cells but inhibited the migratory ability through downregulation of MMP-2 and -9 and vimentin expressions [45]. The other study showed that the concentration of SHK between 2.5 and 10 *μ*g/mL would suppress the proliferation of SMMC-7721, HeLa and HepA_22_ tumor cells [21]. Similar results also revealed that when A549 cells were treated with SHK for 24 hours, 8.0 *μ*M (around 2.67 *μ*g/mL) SHK significantly inhibited cell proliferation, while less than 2.0 *μ*M (around 0.67 *μ*g/mL) SHK significantly suppressed cell adhesion to the extracellular matrix, invasion, and migration [46]. More recently, Lan et al. indicated that the low dose of SHK (1 *μ*M) changed the A549 lung cancer cell number and morphology, while higher doses (4 and 8 *μ*M) of SHK induced cell cycle arrest and apoptosis more significantly [47]. Our results also illustrate this distinct, dose-related growth retardation and apoptotic effects of SHK in lung cancer cells. It depicted a significant increase in apoptosis of A549 cells treated with SHK at 1–2.5 *μ*g/mL concentrations but higher dose (5.0 and 10.0 *μ*g/mL), which on the other hand directly result in both apoptotic and necrotic cell death.

In terms of* in vivo* research, one animal study demonstrated that the median survival time of tumor-bearing mice treated with three different dosages of SHK (2.5, 5.0, or 10.0 mg/kg/day) for 10 days would be prolonged at least for 7 days compared with the sham-treated negative control groups and exhibit hair with a healthier appearance and be more active compared with the 5FU-treated positive control groups [21]. The other study also indicated that SHK treatment at 4.0–8.0 mg/kg/day for 7 days caused 45–60% of tumor growth inhibition by inhibition of proteasome [28]. Of note, it also found that high-dose treatment of SHK (8 mg/kg) would induce adverse toxicity in those mice.

Clinically, SHK would be administered over a protracted period and its chronic toxicity is also important. Previous clinical trial examined in 19 late-stage lung cancer patients revealed that SHK could reduce lung cancer growth effectively (25% in diameter), enhance patient's appetite, preserve body weight, and increase the one-year survival rate (43.3%) via increasing serum interleukin-2 level to improve the immune function [31]. These dose-dependent differential properties might be a therapeutic advantage of this drug over others when encountering different clinical situations.

### 4.2. Apoptosis-, Necrosis-, and Cellular Senescence-Promoting Actions of SHK on Lung Cancer Cells

Apoptosis and necrosis represent the essential mechanisms through antitumor agent work [5, 40]. Treatment with SHK has been reported to cause cell death in various cancer cell lines. Wu et al. demonstrated that SHK would induce about 35, 45, 40, and 15% apoptotic cell death and 15, 20, 30, and 60% of necrotic death in A375-S2 cells by treatment with 5, 10, 20, and 40 *μ*M (around 1.67, 3.3, 6.67, and 13.3 *μ*g/mL) for 24 hours [32]. Piao et al. indicated that incubation for 6 hours of low dose of SHK (1 *μ*M) would induce over 50% of apoptosis in U937 cells, while high dose of SHK (10 *μ*M) provokes necrosis instead of apoptosis [42]. To evaluate the potential effects of SHK against lung cancer cells, five doses of SHK (0.5, 1.0, 2.5, 5.0, and 10.0 *μ*g/mL) were administrated to A549 cells for 24 hours. In accordance with these reports, our results revealed that lower doses of SHK significantly increased early and late apoptosis (16.3%, 36.0%, and 32.1% at doses of 1.0, 2.5, and 5.0 *μ*g/mL), whereas higher doses induced necrosis significantly (26.6% and 47.1% at doses of 5.0 and 10.0 *μ*g/mL). We speculate that higher doses of SHK cause earlier induction of apoptosis, and then necrotic cells increased. Specifically, apoptosis induced by lower doses of SHK occurred later than apoptosis induced by higher doses.

For lung cancer cells, SHK has been specifically shown to cause death of these cells via dysregulation of the ubiquitin-proteasome pathway, activation of caspases, and inhibition of cyclooxygenase-2 [28, 29, 32, 47, 48]. Prior studies reported that SHK induced p53 expression and cell cycle arrest in the G_2_/M phase and prevent G_1_ transition to S phase, suggesting the role of shikonin as DNA-binding cytotoxic agent [23, 32, 49]. The agent, pifithrin-*α*, was a selective p53 inhibitor provided to investigate the chemotherapeutic effect of phytochemical on p53-dependent proapoptotic pathways in A549 cells. Their results demonstrated that pretreatment of pifithrin-*α* significantly inhibited berberine-induced apoptosis in a dose-dependent manner [50]. Our data also showed that the levels of p53, p16, and cleaved caspase-3 significantly increased at 24 hours after the induction of SHK (doses at 1.0 and 2.5 *μ*g/mL); at this time, the release of cytochrome *c* from mitochondria to cytosol was also observed. Interestingly, induction of apoptosis by SHK was inhibited by pretreatment of p53 inhibitor at least partially through coordinative modulation of p16, release of cytochrome *c*, and subsequent activation of caspase-3 cleavage in human lung cancer cells.

Cellular senescence is an aging phenomenon that is characterized by distinct morphological and biological changes [38]. Drugs that intervene in activity of telomerase, the endogenous exclusive protector of telomere, therefore have the potential to act by this novel mechanism to suppress tumor cell proliferation and treat cancer disease [6, 51, 52]. Aoshiba et al. indicated that when A549 cells were treated with 50 *μ*g/mL of bleomycin for 120 hours, they exhibited a typical morphology of cellular senescence characterized by an increased SA-*β*-Gal activity which is a well-established biomarker of senescence [6]. Further, Luo et al. suggested that lower doses of resveratrol treatment (10–50 *μ*M) inhibit lung cancer cell growth by the induction of premature senescence through ROS-mediated DNA damage in a dose-dependent manner [7], while higher doses (100–200 *μ*M) may induce apoptosis in tumor cells. In accordance with these reports, our data demonstrated similar effects of SHK on lung cancer cells in that SHK at lower doses (0.5–2.5 *μ*g/mL) triggered A549 cells to present a senescence phenotype characterized and higher doses (5–10 *μ*g/mL) apoptosis and even necrosis. These effects of SHK on positive-SA-*β*-Gal staining in A549 cells suggested that lower concentration of SHK would induce senescence, while higher doses enhance apoptosis and even necrosis in lung cancer cells. Additionally, lung cancer cells, when exposed to SHK (1–5 *μ*g/mL), were subjected to senescence and apoptosis along with elevated caspase-3 activity and expression of cell cycle regulators (p53 and p16) and cytochrome *c* release to cytosol.

SHK might have multiple cellular targets in order to achieve their biological beneficial effects for tumor growth inhibition. The findings that SHK could modify the expression of p53 [46, 53, 54] and the downstream effectors p16 [55], a positive regulator of retinoblastoma protein that causes senescence of tumor cells [56], suggest that SHK could behave as other antineoplastic agents to inhibit tumor cell growth. A “blueprint” of the proteins and pathways necessary for the cytotoxic action of a given drug, SHK, coupled with an understanding of the molecular basis of differential dose-related drug behaviors, may provide the necessary information to tailor existing therapies for individual tumors. Nevertheless, A549 cells appear sensitive to SHK treatment; however, the exact mechanistic basis for which is outside the scope of the current paper and the potential use of SHK for the treatment of lung cancer should be guided by the understanding that its mechanism of action is similar to a number of well-established cytotoxic chemotherapeutic agent.

Though previous animal studies [21, 28, 30] and initial human trial [31] indicated that SHK is a relatively safe compound with no adverse side effects, further research is needed to confirm the potential mechanisms of SHK through p53 regulated signaling actions on apoptotic and necrotic inductions in human lung cancer xenograft mouse model for developing more effective strategies for cancer treatment* in vivo*. In addition, randomized controlled trial is also desired to investigate the efficacy and safety of SHK for lung cancer therapy in patients.

## 5. Conclusion

Overall, it was the first report showing that SHK could exert diverse growth inhibitory actions on A549 lung cancer cells promoting cellular senescence, apoptosis, and necrosis in a dose-related continuum. Thus, SHK has a great potential in inducing various forms of death of lung cancer cells and may play a promising role in chemotherapeutic treatment of such a malignant disease.

## Figures and Tables

**Table 1:** The half maximal inhibitory concentration (IC_50_) of DOX, BLM, and SHK in A549 cells by MTT assay.

MTT assay	DOX	BLM	SHK
Test range (*μ*g/mL)	0.5–10	0.5–10	0.5–10
Linear range (*μ*g/mL)	0.5–10	0.5–10	0.5–10
Repression equation	*Y* = 0.1682*x* + 1.0627	*Y* = 0.0960*x* + 1.1074	*Y* = −0.1836*x* + 1.1633
*R* ^2^	0.8559	0.9778	0.9691
IC_50_ (*μ*g/mL)	0.49	6.19	3.52
95% CI of IC_50_ (*μ*g/mL)	0.47–0.52	5.88–6.50	3.34–3.69

IC_50_: the half maximal (50%) inhibitory concentration.

CI: confidence interval.

## Abbreviations

SHK: Shikonin
BLM: Bleomycin
DOX: Doxorubicin
ATCC: American type culture collection
DMEM: Dulbecco's modified Eagle's medium
FBS: Fetal bovine serum
BSA: Bovine serum albumin
DMSO: Dimethyl fulfoxide
PI: Propidium iodide
MTT: 3-(4,5-Dimethylthiazol-2-yl)-2,5-diphenyl-tetrazolium bromide
FITC: Fluorescein isothiocyanate
FACS: Fluorescence-activated cell sorting
SA-*β*-Gal: Senescent associated-*β*-galactosidase
DAPI: 4′,6-Diamidino-2-phenylindole.
